# Editorial: Respiratory support strategies in the prevention and treatment of BPD: many questions, few answers

**DOI:** 10.3389/fped.2023.1233810

**Published:** 2023-07-12

**Authors:** Huayan Zhang, Robin L. McKinney

**Affiliations:** ^1^ Division of Neonatology and Center for Newborn Care, Guangzhou Women and Children’s Medical Center, Guangzhou, China; ^2^ Division of Neonatology, Children’s Hospital of Philadelphia and University of Pennsylvania Perelman School of Medicine, Philadelphia, PA, United States; ^3^ Department of Pediatrics, Alpert Medical School of Brown University, Providence, RI, United States

**Keywords:** bronchopulmonary dysplasia, pulmonary hypertension (PAH), chronic mechanical ventilation, non-invasive respiratory support (NIRS), chronic lung disease of prematurity

**Editorial on the Research Topic** Respiratory support strategies in the prevention and treatment of bronchopulmonary dysplasia

With increased survival of premature infants, bronchopulmonary dysplasia (BPD), has become the major cause of chronic lung disease in children. It confers significant morbidity, such as chronic cardiopulmonary insufficiency, growth failure and neurodevelopmental delay, and an increased burden on the health care system and society. Since its first description by Northway and colleagues in 1967 (1), understanding the pathophysiology has allowed us to develop management techniques that should mitigate the development of BPD, but despite this the incidence of BPD has not changed for decades (2). As a mainstay therapy for preterm infants with respiratory insufficiency, there have been a series of advancements in the respiratory management of preterm infants. However, much more needs to be learned about how to apply these therapies to prevent the development of BPD and/or treat infants with established BPD. With this realization, we proposed this research topic and gathered a team of neonatal and pediatric specialists to examine ways to predict, prevent, and manage BPD once developed.

## Can we reliably predict the risk of BPD or BPD-associated pulmonary hypertension?

Given that BPD carries with it significant morbidity in survivors, early recognition allows the clinician opportunity to council families. Many risk factors for the development of BPD have been discovered and explored, some more pertinent than others. Some variables such as small for gestational age (SGA) and birth weight are well known, but they are not the only contributing factors. It is important to take the larger clinical picture into account which, given the number of possible variables, is overwhelming at the bedside. To simplify things for the clinician, Yin et al. provide a predictive model based off seven variables that can be used to calculate the risk of BPD at day 14 of life (Yin et al.). Such models are useful not only for counseling parents, but also for designing studies to see if early interventions focused on identified risk factors can prevent BPD. In addition to predicting the risk of developing BPD, some morbidities such as BPD associated pulmonary hypertension (BPD-PH), carry a particularly high rate of mortality. Predicting which infants are at high risk of developing BPD-PH is an active area of research, asking such questions as who is at risk of developing BPD-PH, and when and how often should we screen infants at risk of BPD? Local practices and small sample sizes at individual centers make teasing out which variables are important difficult as demonstrated by two of the articles published in this research topic; one which demonstrates the presence of a hemodynamically significant PDA longer than 28 days is a risk factor and another article which showed that surgical closure of a PDA is also a risk (Wang et al., Chang et al.). At first glance these appear to be opposite conclusions surrounding the management of PDAs, but may represent the uncertainty regarding the optimal time for closure of a PDA and are indicative of the challenges facing both bedside clinicians and researchers. Further, most of the research that has been conducted is retrospective in nature and focused on BPD prevention with significantly fewer studies examining what to do once BPD has developed. However, with advancement in chest imaging techniques, combining imaging with clinical characteristics might be helpful in predicting and/or guiding management of infants developing BPD-PH as demonstrated by Yao et al.

## Do we know the best mode of non-invasive respiratory support for BPD prevention?

Mechanical ventilation is a known risk factor for the development of BPD where non-invasive ventilation has been shown to be protective. But beyond these broad categories is there any technique that leads to better outcomes? Is there an ideal mode of non-invasive ventilation in the prevention of BPD? Neither Wu nor Alvila-Alvarez with vastly different studies were able to show that one modality of non-invasive ventilation influenced the development of BPD over another (Avila-Alvarez et al., Song et al.). However, Hysinger and Ahlfeld pointed out in their review that prolonged constant distending pressure might be helpful, especially in extremely preterm infants (Hysinger and Ahlfeld). Given the heterogeneity within patients and multiple risk factors identified, might there be subsets of patients that would respond to different interventions? Close examination of risk factors will inform the design of future studies that will help answer these questions.

## What are the ideal mechanical ventilation strategies for BPD prevention and/or management?

What about infants in whom mechanical ventilation cannot be avoided after birth? In their review, Hysinger and Ahlfeld emphasized the utility of open lung ventilation in avoiding both atelectotrauma and volutrauma with a goal of supporting the cardiopulmonary needs of the extremely preterm infants while minimizing lung injury (Hysinger and Ahlfeld). In addition to existing literature demonstrating the benefit of volume targeted ventilation (3), two newer studies published in this research topic further supported the use of open lung ventilation in BPD prevention. In a retrospective cohort study, Guaman et al. found that the use of volume guarantee ventilation (VGV) is associated with a decreased risk of BPD when compared with pressure-limited ventilation (PLV) (Guaman et al.). In the other retrospective observational study, proactive ventilator adjustment was performed in infants at high risk for death and BPD (Sammour et al.). Through providing adequate PEEP and tidal volume, the team attempted to provide adequate respiratory support so that patient-ventilator synchrony is improved, and air trapping was avoided. This early intervention strategy resulted in a decreased need for tracheostomy and decreased length of stay in their unit. Although retrospective and single center in nature with small sample sizes, the investigators of these studies attempted to address an important topic, that is, how to best handle infants on a ventilator who are “developing” BPD. These investigators also brought up the importance of lung mechanics-based precision ventilator management. Further, Stockard et al. reinforced the concept of precision medicine by demonstrating that pharmacometabolomic profiling may help differentiate infants who may respond to dexamethasone therapy from those who may not (Stockard et al.).

Unfortunately, there are even less high-quality evidence to guide the respiratory management of infants who have already developed BPD. In this research topic, several review articles provided comprehensive reviews of currently available evidence (Hysinger and Ahlfeld, Miller et al., Logan et al.). The authors advocated for a pathophysiological-based ventilator management approach and emphasized that “the consistent provision of adequate support is fundamental” to the management of infants with established and longstanding BPD. Further, Akangire and Manimtim made an effort to address the questions of the indication and timing of tracheostomy placement, timing of liberation and outcomes in infants with severe BPD (Akangire and Manimtim). Their review highlighted significant center variability in both intensive care and out-patient follow-up due to the lack of high-quality evidence.

In summary, many questions remain to be answered and multicenter prospective studies focusing on respiratory support strategies in the prevention and treatment of BPD are needed to guide clinical care and decrease variation in the care of these infants.
